# Pregnancy in Glomerular Disease: From Risk Identification to Counseling and Management

**DOI:** 10.3390/jcm13061693

**Published:** 2024-03-15

**Authors:** Veronica Maressa, Elisa Longhitano, Chiara Casuscelli, Silvia Di Carlo, Luigi Peritore, Domenico Santoro

**Affiliations:** Unit of Nephrology and Dialysis, Department of Clinical and Experimental Medicine, Azienda Ospedaliera Universitaria “G. Martino”, University of Messina, 98124 Messina, Italy; elisa.longhitano@libero.it (E.L.); chiara.casuscelli88@gmail.com (C.C.); silviadicarlo1@yahoo.it (S.D.C.); luigiperitore1994@gmail.com (L.P.); domenico.santoro@unime.it (D.S.)

**Keywords:** pregnancy, glomerulonephritis, counseling, kidney, glomerular disease

## Abstract

**Background**: Pregnancy involves complex hemodynamic and immune adaptations to support the developing fetus. The kidney assumes a pivotal role in orchestrating these mechanisms. However, renal disease poses a potential risk for adverse maternal–fetal outcomes. While kidney function, hypertension, and proteinuria are recognized as key influencers of risk, the mere presence of glomerular disease, independent of these factors, may wield significant impact. **Methods**: A brief review of the existing literature was conducted to synthesize current knowledge regarding the interplay between glomerulonephritis and pregnancy. **Results**: The review underscores the centrality of the kidney in the context of pregnancy and highlights the role of glomerular disease, particularly when active. It emphasizes multifaceted risk modulators, including kidney function, hypertension, and proteinuria. **Conclusion**: Understanding the dynamics between pregnancy and glomerulonephritis is crucial for optimizing maternal and fetal outcomes. Preconception counseling and collaborative nephro-gynecological management emerge as pivotal components in addressing the unique challenges posed by this medical interplay.

## 1. Introduction

Due to the kidney’s crucial role in adapting to pregnancy, it is unsurprising that changes in renal function may correlate with adverse maternal–fetal outcomes. Studies indicate a decline in outcomes with chronic kidney disease (CKD), starting in the early stage and worsening proportionately to the level of renal impairment [1,2,3,4]. Regardless of renal function, hypertension and proteinuria are acknowledged as influencers of pregnancy outcomes [1,2,3,5,6,7]. Conversely, the standalone impact of glomerular disease remains unclear. This concise review seeks to synthesize existing literature on maternal–fetal outcomes for individuals with glomerulopathy, exploring the interplay between the disease and pregnancy, as well as the reciprocal influence of pregnancy on the disease.

## 2. Kidney Function in Physiological Pregnancy

The kidney undergoes structural and functional modifications starting from the early weeks of pregnancy to ensure adequate uteroplacental perfusion without compromising maternal perfusion and to facilitate optimal fetal development [8,9]. Key structural changes, primarily driven by combined hormonal and mechanical factors, include hydronephrosis and an increase in total renal volume, predominantly attributed to elevated vascular volume [9,10,11,12]. Furthermore, the hormonal changes in pregnancy induce vasodilatation and relative resistance to vasoconstrictors, leading to glomerular hyperfiltration [8,9]. This hyperfiltration, in turn, results in reduction of serum creatinine and BUN and in increased proteinuria, with levels considered normal down to <300 mg/24 h [8,13,14,15]. Additionally, the interaction among mineralocorticoids, progesterone, anti-natriuretic peptides, and natriuretic peptides causes the retention of sodium and potassium [8,9,16]. Importantly, this retention does not translate into increased serum concentrations due to dilution resulting from the reduced threshold of ADH secretion and the consequent rise in the reabsorption of free water [17,18].

## 3. Materno-Fetal Outcomes in Women with a Known Glomerular Disease

There exists a heightened vulnerability to adverse events during pregnancy in women with a glomerular disease. The available data show that renal function, hypertension, and proteinuria emerge as linchpin determinants [1,2,3,5,6,7]. Concerning renal function, women without alteration before pregnancy show no significant increase in the risk of renal function deterioration during or after pregnancy; in contrast, individuals with preexisting kidney function alterations are predisposed to experience a decline in renal function [1,2,3,4,19,20,21,22,23,24,25]. While maternal mortality remains infrequent, the risk escalates in cases of severe exacerbations of immunological diseases, particularly when active at the pregnancy’s beginning [26]. Hypertensive disorders, including preeclampsia (PE), are more frequent, but they are pervasive among women with varied kidney diseases, transcending the confines of glomerular disease [5,26,27].

Offspring risks, notably preterm delivery, and intrauterine growth restriction, extend beyond the exclusive domain of preeclampsia and glomerular disease to encompass the broader spectrum of kidney diseases [2,5,26]. The heightened risk of intrauterine demise is particularly evident in women with lupus nephritis, diabetic nephropathy, and other systemic immunologic disease [5,26].

### Risk of Preeclampsia and the Role of Biomarkers

There is an intricate and bidirectional relationship between glomerulonephritis (GN) and the risk of developing PE [27]. As proposed by Redman et al., PE pathogenesis involves abnormal uterine spiral artery formation, leading to placental oxidative stress and endothelial injury [28]. This model is supplemented by a second placental etiology involving altered angiogenic factors [29]. Notably, the role of vascular endothelial growth factor, placenta growth factor, soluble fms-like tyrosine kinase-1, and endoglin in the imbalance of angiogenic factors is crucial. The kidney, undergoing adaptive changes during normal pregnancy, becomes vulnerable in the presence of conditions like renal disease, chronic hypertension, autoimmune disorders, and diabetes, influencing multiple aspects of placentation [27,30,31].

An enduring misconception suggests that preeclampsia (PE) predominantly occurs in first pregnancies. However, data indicate a more nuanced scenario, demonstrating a conditioned probability of PE incidence in subsequent pregnancies. A comprehensive study conducted by Hernandez Diaz and colleagues in Sweden in 2009 highlighted that while the risk of PE in first pregnancies stood at 4.1%, the incidence decreased to 1.7% in subsequent pregnancies for women without prior PE [32]. Conversely, experiencing PE in the first pregnancy significantly increased the risk of recurrence in subsequent gestations, reaching 14.7% and escalating to 31.9% after two PE episodes.

While these trends hold for singleton pregnancies, the risk of initial PE is higher in multiple pregnancies, estimated at 12% in the Swedish study [32]. However, this heightened risk does not significantly impact the recurrence of PE in subsequent singleton pregnancies. Additionally, the phenotype of PE appears to modulate the risk of recurrence, with “early-onset” PE associated with substantially higher recurrence rates [32].

Although data on recurrence risk vary, with some studies reporting lower rates of PE recurrence, recent meta-analyses have identified additional maternal predisposing factors for PE onset [33]. These include a personal or familial history of hypertensive disorders, obesity, advanced maternal age, chronic hypertension, pre-existing diabetes, and autoimmune diseases, such as antiphospholipid syndrome and systemic lupus erythematosus, which share similar risk profiles with chronic kidney disease (CKD) [33,34].

Furthermore, in addition to multiple pregnancies, the utilization of assisted reproductive technology warrants closer monitoring during gestation, particularly in cases involving egg donation, which may be associated with increased maternal age and decreased baseline kidney function [35].

In IgA nephropathy, women exhibit a heightened risk of PE, which is potentially attributed to elevated soluble fms-like tyrosine kinase-1 levels [27]. Focal segmental glomerulosclerosis is strongly linked with PE, with hyperfiltration stress identified as a potential risk factor [27]. Lupus nephritis increases the risk of PE, and distinguishing between LN and PE remains a challenge for nephrologists [27]. Women with diabetic nephropathy face increased rates of hypertensive disorders during pregnancy, with PE prevalence reaching 35–64% [27].

Preeclampsia superimposed on glomerular disease typically unfolds late in pregnancy, often intertwined with well-preserved fetal growth [26,27]. Conversely, in PE without kidney disease, kidney damage is characterized by endotheliosis and podocytopathy [30].

The identification of novel biomarkers for early detection of preeclampsia is paramount.

In line with the theory that placental damage precedes endothelial dysfunction, molecules originating from the placenta are potential rational biomarkers [36].

Studies have demonstrated alterations in placental mRNA, miRNA, long-noncoding RNA, and circular RNA in comparing healthy and preeclamptic women [37,38,39].

While the measurement of circulating proteins may offer more consistency than RNA species, abnormal levels of placental protein 13 (PP13), pregnancy-associated plasma protein A (PAPP-A), Alpha-fetoprotein (AFP), and growth differentiation factor 15 (GDF-15), rather than placental growth factor (PIGF) alone or the soluble fms-like tyrosine kinase 1/placental growth factor (sFlt-1/PIGF) ratio, are associated with adverse pregnancy outcomes, including preeclampsia [40,41].

Like the placenta, endothelial cells release miRNAs; notably, Whigham et al. underscored decreased levels of GATA2 and miR126 [42].

Despite their modest predictive efficacy, recent meta-analyses have demonstrated alterations in preeclampsia patients’ asymmetric dimethylarginine (ADMA) values [43,44].

Some studies have shown a two- to threefold increase in endothelin-1 values, which is a potent vasoconstrictive peptide, in pregnancies with preeclampsia [45,46].

Additionally, in a small cohort, the soluble form of VCAM-1, a vascular adhesion molecule, was found to be upregulated in response to endothelial inflammation in early-onset preeclampsia [47].Unfortunately, these biomarkers have not yet been validated, and in clinical practice, only the sFlt1/PlGF ratio is utilized almost exclusively and not universally.

## 4. Pregnancy and Primary Glomerulonephritis

The available data suggest that IgA nephropathy (IgAN) is the most common glomerulopathy during pregnancy, while focal segmental glomerulosclerosis (FSGS) is associated with poorer outcomes, and membranous nephropathy (MN) tends to have more favorable results [5].

Table 1 summarizes the main outcomes from studies with more than one case described, published since 2008.

In summary, concerning IgA nephropathy (IgAN), recent studies have indicated that pregnancy does not hasten the deterioration of renal disease in normotensive women with IgAN and mild CKD (GFR > 70 mL/min/1.73 m^2^). However, a different scenario emerges for patients with more severe renal impairment (GFR < 70 mL/min/1.73 m^2^), those with other factors influencing adverse pregnancy outcomes (such as hypertension), or those experiencing a complicated pregnancy (e.g., preeclampsia (PE)), wherein the risk of renal disease progression is elevated [48,49,50,51,52,53,54,55,56,57,58,59].

In the context of podocytopathies, for pregnancies in women with FSGS, although a decline in neonatal mortality among old and new studies (probably for improved maternal and neonatal care), persisting high risks for miscarriage, preterm birth, and small for gestational age (SGA) linked to complicated pregnancies [49,52,58,60,61,62,63,64]. Limited data exist on long-term renal function; however, studies have suggested a higher incidence of renal replacement therapy in women with FSGS after 10 years compared to those without a history of pregnancy (19–22% vs. 7%) [60,64]. Unfortunately, in this case, the role of PE as “second hit” in contributing to CKD progression remains unclear [65].

In the studies in the context of minimal change disease (MCD), the role of risk modulators is once again emphasized [58,62,66,67,68]. Indeed, in 1984, Surian documented two pregnancies in MCD-afflicted women who, devoid of gestational hypertension or proteinuria, successfully carried the pregnancies to term without complications [58]. Conversely, in 1985, Abe et al. reported complications (including SGA, premature deliveries, neonatal deaths, and miscarriage) in a cohort of MCD-affected women, with 5.9% experiencing hypertension and 5.9% exhibiting proteinuria [68]. Recent findings by O’Shaughnessy et al. highlighted five of seven cases involving preterm delivery in women with MCD, of which 28.6% displayed proteinuria [62].

Membranous nephropathy (MN) appears to be associated with more favorable maternal–fetal outcomes, although adverse outcomes such as neonatal deaths, miscarriage, preterm delivery, SGA, renal deterioration, and PE are reported. However, also within the MN context, higher rates of adverse maternal–fetal outcomes have been observed in pregnancies complicated by hypertension and/or proteinuria [49,58,61,62,63,66,67,68,69,70,71,72,73]. In addition, recent findings by Liu et al. highlight associations among hypoalbuminemia, lack of disease remission, PLA2R positivity, and adverse maternal–fetal outcomes [72].

Finally, in the context of membranoproliferative glomerulonephritis (MPGN), limited previous studies have revealed variable rates of neonatal death, miscarriages, preterm births, and SGA [49,58,67]. Once more, the presence of risk modulators significantly influenced maternal–fetal outcomes, as underlined by Barcelò et al., who highlighted that women with MPGN, particularly those with proteinuria >2.5 g/24 h, hypertension, and impaired renal function, faced a higher risk of adverse outcomes during pregnancy [49].

**Table 1:** Summary of studies reporting the outcome of pregnancy in patients with primary glomerulonephritis published since 2008.

Study/Yr	Study Characteristics	Pregnancies	Creatinine before Pregnancy or at Conception (mg/dL)	Proteinuria before Pregnancy or at Conception (g/d)	Patients with Pregestational Hypertension—n (%)	Neonatal Death—n (%)	SGA/IUGR—n (%)	Preterm Delivery—n (%)	PE—n (%)
IgAN									
Limardo et al. (2010) [53]	Multicentric longitudinal cohort study	229	0.87 ± 0.15	1	27 (20%)	1 (0.4%)	22 (11%)	20 (10%)	17 (9%)
Shimizu et al. (2010) [57]	Prospective follow-up study	29	0.68 ± 0.10 (*n* = 5) 0.75 ± 0.05 (*n* = 16) 0.94 ± 0.21 (*n* = 8)	NA 0.39 ± 0.22 0.77 ± 0.31	NA	0	0	0	0
Waness et al. (2010) [59]	Prospective study	12	0.88	0.5	2 (17%)	0	0	0	3 (25%)
Oh et al. (2011) [74]	Single-center retrospective study	52	0.8 (0.5—2.6)	0.7 g/g	25 (48%)	NA	13 (25%)	8 (15%)	NA
Liu et al. (2014) [54]	Matched-cohort study	69	eGFR 102 mL/min	1.27 (0.06–7.25)	7 (11%)	NA	8 (14%)	7 (12%)	6 (9%)
Shimizu et al. (2015) [75]	Prospective follow-up study	9 (eGFR ≥ 45 mL/min) 7 (eGFR < 45 mL/min)	0.98 ± 0.1 1.3 ± 0.1	1 ± 0.8 1.3 ± 0.4	NA	0	3 (19%)	0	NA
O’Shaughnessy et al. (2017) [62]	Retrospective study (Glomerular Disease Collaborative Network registry and the UNC Hospitals Perinatal Database)	18	1 (0.8–1.2)	1.3 (0.9–4.1)	3/15 (20%)	2 (11%)	2 (11%)	6 (33%)	6 (33%)
Su et al. (2017) [76]	Matched-cohort study	116	eGFR 102.6 ± 23.9 mL/min	1.04 (0.03–7.25)	15 (14%)	1 (0.9%)	16 (14%)	13 (11%)	12 (10%)
Piccoli et al. (2017) [77]	Multicentric cohort study	33	0.87 (0.5–2.88)	>0.5 in 17 women (53.1%)	9 (27%)	NA	10 (30%)	12 (36%)	4 (17%)
Park et al. (2018) [78]	Retrospective study (propensity-score-matched cohort Analysis)	31 (eGFR > 90 mL/min) 21 (90 < eGFR ≤ 60 mL/min) 12 (eGFR < 60 mL/min)	0.63 (0.60–0.69) 0.90 (0.82–0.92) 1.60 (1.23–1.86)	NA	12 (40.0) 14 (66.7) 9 (75.0)	NA	16 (30%)	21 (39%)	13 (24%)
Jarric et al. (2021) [50]	Retrospective study (Register-based cohort study)	327	NA	NA	28 (8.6%)	0	53 (16%)	43 (13%)	45 (14%)
Tang et al. (2021) [79]	Retrospective study (reviewed medical records)	63	eGFR 93 mL/min	0.8	9 (14%)	NA	11 (18%)	16 (25%)	14 (22%)
MCD									
O’Shaughnessy et al. (2017) [62]	Retrospective study (Glomerular Disease Collaborative Network registry and the UNC Hospitals Perinatal Database)	7	0.7 (0.5–0.7)	0.3 (0.1–0.3)	0	0	0	5	2
FSGS									
De Castro et al. (2017) [63]	Retrospective study (chart review at a single tertiary center)	12	0.4–1	NA	NA	0	NA	NA	NA
O’Shaughnessy et al. (2017) [62]	Retrospective study (Glomerular Disease Collaborative Network registry and the UNC Hospitals Perinatal Database)	17	1 (0.8–1.8)	2.4 (1.2–4.1)	11/16 (69%)	0	4 (24%)	10 (59%)	8 (47%)
Attini et al. (2017) [80]	3 cases report	3	0.6 0.8 0.84	2.1 3.03 6.3	NA	0	1 (33%)	0	0
Guillen et al. (2019) [81]	3 cases report	3	NA	NA	NA	0	2/2(100%)	2/2 (100%)	1/2 (50%)
MN									
De Castro et al. (2017) [63]	Retrospective study (chart review at a single tertiary center)	4	NA	NA	NA	NA	NA	NA	NA
O’Shaughnessy et al. (2017) [62]	Retrospective study (Glomerular Disease Collaborative Network registry and the UNC Hospitals Perinatal Database)	6	0.8 (0.6–0.9)	5.3 (1.1–8.3)	3 (75%)	0	0	2 (33%)	0
Liu et al. (2020) [72]	Retrospective study	27	0.71 (11/27 pregnancies)	0.6 ± 0.6 (11/27 pregnancies)	2 (7%)	0	NA	7 (26%)	4 (15%)

Legend: NA, not available; eGFR, estimated glomerular filtration rate.

## 5. Lupus Nephritis and Pregnancy

The changes in the immune system during pregnancy have the potential to trigger flares of systemic lupus erythematosus (SLE), particularly in cases in which there is kidney involvement [82,83,84,85,86].

Studies highlight that pregnant women with lupus nephritis (LN) have an increased risk of complications, including gestational hypertension, flares, irreversible kidney damage, and PE [84,85,86,87,88]. The risk of maternal mortality (20-fold increased) and various complications, such as thrombosis, thrombocytopenia, infection, and the need for transfusion (three- to sevenfold increased), is significantly elevated in women with SLE during pregnancy in a study published in 2008 [89]. Factors such as proteinuria (especially if >1 g/24 h), low GFR (mainly if <60 mL/min), low C3 levels, anti-DNA antibody positivity, and high body mass index contribute to flares and worsening kidney injury [86,90].

Additionally, pregnant women with SLE and renal involvement have a higher risk of spontaneous miscarriage, preterm delivery, neonatal death, intrauterine growth restriction (IUGR), and SGA compared to the general population [84,85,91].

In the cases of concomitant antiphospholipid syndrome (APS), which often complicates SLE pregnancies, IUGR, SGA, preterm delivery, and miscarriage are more frequent, as well as PE, eclampsia, and HELLP syndrome (hemolysis, elevated liver enzymes, and low platelets) [92,93].

In addition to the other fetal complications, neonatal lupus syndrome, although rare, can occur, particularly in the presence of positive maternal antibodies to intracellular ribonucleic proteins for Sjögren’s syndrome type A antigen (SSA) and Sjögren’s syndrome type B antigen (SSB), which are transported across the placenta [94]. The main manifestations are cutaneous, hematological, and cardiac damage, such as congenital heart block [94].

## 6. Diabetic Nephropathy and Pregnancy

There are many studies in the literature on pregnancy outcomes in women with pregestational diabetes, but there are few inconsistent data on pregnancy outcomes in women with diabetic nephropathy (DN) [31]. Of the available data, it emerges that the occurrence of maternal and fetal complications correlates with glycemic control and severity of chronic disease, with a greater tendency for deterioration in renal function in the presence of pre-pregnancy GFR values <60 mL/min and/or proteinuria >3 g/day [95]. As shown by Carr et al., end stage renal disease (ESRD) is more common in women with uncontrolled hypertension at baseline or during pregnancy [96]. According to a recent analysis of three Norwegian registries, including 2204 women with pregestational diabetes, the risk of ESRD or death was higher in patients with a pregnancy complicated by PE [97]. The latter, in a large Swedish study, has been shown to be more frequent in diabetic type 1 patients compared to non-diabetic control patients (OR 4.47) [98]. Sibai et al. also showed, in a prospective analysis of 462 women with pregestational diabetes, that the frequency of PE was higher in the presence of renal involvement (35–66% vs. 9–24%) [99]. In addition to increasing the risk of progression of renal disease, proteinuria at baseline is a modulator of poor fetal and maternal outcomes, with a higher risk of preterm birth, and SGA [99]. Women with diabetic nephropathy also have an increased risk of gestational hypertension, placental dysfunction, and IUGR. Pregnant diabetic women, finally, also have a higher incidence of miscarriage, congenital malformations, and macrosomia than the general population [100].

## 7. Vasculitis and Pregnancy

Data on pregnancy in patients with vasculitis are very scarce, partly because, except for Takayasu’s disease and Behçet’s disease, vasculitis is rare in women of reproductive age. In addition, in the few patients of childbearing age, multi-organ involvement tends to discourage pregnancy [31]. Risk data are mainly derived from individual clinical cases or, at best, from retrospective studies, often with inherent observational bias. Despite these limitations, it can be argued that the worst maternal–fetal outcomes are associated with the presence of active disease or disease diagnosed during pregnancy [31]. Pulmonary hemorrhage, rapidly progressive glomerulonephritis, polyneuritis, and myocarditis, in fact, have been described as life-threatening complications in women with active relapsing disease or de novo vasculitis in pregnancy [101,102].

Fredi et al., in a multicenter study, followed 65 pregnancies in 50 women with vasculitis one year before and one year after delivery and compared maternal and fetal outcomes in a control population of 3939 mothers [103]. Vasculitis-associated complications occurred in 35.4% of pregnancies, with 7.7% being severe events, including three cases of transient ischemic attack (TIA) [103]. Preterm delivery (<34 weeks) and cesarean section were more common in the vasculitis group than in controls [103]. After delivery, twelve flares occurred, including one serious event [103]. A similar retrospective study published in 2021 analyzed maternal and fetal outcomes in the different classes of vasculitis [104]. Relapse was more common in small vessel vasculitis [104]. PE complicated the pregnancy in women with large- and small-vessel disease, whereas IUGR occurred in only one case of small-vessel vasculitis [104]. In the latter case, a recent meta-analysis showed that the most common complications were preterm birth, IUGR, and disease flare-ups [105].

## 8. Counseling and Management

Effectively managing pregnancy in individuals with glomerulopathy demands a thorough approach that pays attention to various medical considerations. A cornerstone of this approach is pre-pregnancy counseling [5,31,106,107,108]. At this pivotal stage, women must be educated not only about the heightened risks associated with their condition but also about the potential for a successful pregnancy, particularly when tailored interventions are applied.

A recent survey of Australian women with CKD and their partners or family members concluded that women’s information needs are still unmet, underlining the imperative of the need for training of healthcare professionals and collaboration between nephrologists and gynecologists from the early stages of discovering kidney disease in fertile women wishing to become pregnant [109].

Pre-conception counseling should be accessible to all women with glomerulonephritis. The goal is to guide them toward a planned pregnancy. A critical emphasis should be placed on the necessity of a pre-conception stabilization period lasting at least six months for the glomerular disease [5,31,106,107,108]. During this period, efforts should focus on achieving and maintaining minimal proteinuria levels using permitted immunosuppressive drugs, contributing to a more favorable environment for a successful pregnancy [5,31,106,107,108].

Striking the right balance between preserving maternal health and ensuring fetal well-being is paramount. This may involve a collaborative effort between healthcare professionals from various specialties to devise a comprehensive and tailored management plan.

As suggested by the Italian Best Practices for the care of CKD in pregnancy, the frequency of nephrological follow-up should be at least a control with blood and urine tests every 4–6 weeks in the early stage of CKD, or closer in the advanced stage or in the presence of risk modulators [26,31].

In cases of genetic forms of glomerulopathy, genetic counseling becomes a crucial component of care, providing individuals and their families with insights into the risks of transmission and potential hereditary implications [31,106,107,108].

Given that even mild renal insufficiency increases the risk of hypertensive disorders in pregnancy and that blood pressure >140/90 mmHg negatively impacts fetal survival, blood pressure monitoring and control is essential in pregnant women with glomerulonephritis [26].

While evaluating, the sflt1/PIGF ratio, especially in high-risk cases, is advisable for ensuring timely interventions [31]. Typically, during the aggravation of preexisting CKD, the sflt1/PIGF ratio remains within standard parameters; however, this equilibrium takes an upward turn when preeclampsia is in the picture [26]. Prudence is essential when deciphering variations in this ratio, given that preeclampsia may superimpose itself on CKD, yielding intermediary levels [26]. Severe uteroplacental Doppler flows indicate placental involvement, often associated with intrauterine growth restriction; conversely, declining kidney function without hypertension tends to uphold fetal growth [26].

While renal biopsy is not categorically contraindicated during pregnancy, its consideration should be judicious, particularly when therapeutic decisions hinge on its results.

Postpartum monitoring remains a paramount aspect of the overall care plan, extending well into the first few years after childbirth to address and mitigate potential long-term implications [31,106,107,108].

The topics of immunosuppression, antihypertensive therapy, and some other general indications are deepened in the following paragraphs.

### 8.1. Immunosuppressive Therapy in Pregnancy

Transitioning from potentially teratogenic immunosuppressants to pregnancy-compatible alternatives (Table 2) is a delicate process that requires careful consideration [31,110].

Solely considering drug safety is insufficient. This is not only because a margin of uncertainty persists even for commonly deemed safe drugs like steroids (with a risk of increased palatoschisis and transient neonatal immunodepression, the latter being dosage-related) or calcineurin inhibitors (risk of under- or overdosing linked to changes in distribution volume and the percentage of active drug) but also because the treatment risk must be weighed against the risks associated with exacerbations and recurrences of renal disease [110]. Risks are, however, “certain” for drugs like mycophenolate mofetil, cyclophosphamide, methotrexate, and mTOR inhibitors, which are responsible for fetal malformations, and therefore, they should be replaced with non-teratogenic drugs before planning a pregnancy [110].

Concerning the optimal switch between immunosuppressive drugs during pregnancy, the change from mycophenolate mofetil (MMF) to azathioprine (AZA) before pregnancy is suggested by current guidelines, although the precise timing of the switch is not clearly defined. It is recommended to discontinue MMF use at least six weeks before conception in women with kidney transplants and three months before conception in women with lupus nephritis [111]. However, some authors suggest a longer period up to six months before pregnancy [112].

Regarding tacrolimus and cyclosporine, international guidelines and Italian best practices do not recommend changing during pregnancy [113,114].

mTOR inhibitors, such as sirolimus and everolimus, require precautionary discontinuation before pregnancy (twelve weeks for sirolimus and eight weeks for everolimus) [113,115].

Cyclophosphamide poses higher risks when the fetus is exposed to the drug in the first trimester, and because of this, it must be discontinued before pregnancy. Conversely, risks are lower with exposure in subsequent trimesters (second and third trimesters), and some authors suggest considering its use in potentially life-threatening autoimmune diseases and using cyclophosphamide during the second and third trimesters of gestation, balancing the risks associated with preterm birth with the damage associated with fetal exposure [116].

Many experts advise against the use of rituximab before and during pregnancy due to the risk of neonatal immunosuppression. The ideal time for discontinuation has not been identified, although a 6-month interval is typically recommended in line with the median time commonly recommended for reinjections. However, rituximab may be continued conditionally, with monitoring of neonatal B cells, during attempts to conceive and pregnancy, according to the latest guidelines from the American College of Rheumatology [114].

Eculizumab is usually suspended during pregnancy due to insufficient data on its use in this context [117].

Hydroxychloroquine is considered safe and recommended for patients with systemic lupus erythematosus [111].

Table 3 outlines the permissible treatment options for various glomerulonephritis during pregnancy.

Unfortunately, the ideal scenario (a stable disease with drugs allowed in pregnancy) does not always materialize. The likelihood of unplanned pregnancies remains significant, often due to unawareness of kidney disease, the diagnosis of which only emerges when pregnancy complications arise. Furthermore, there is a lack of experience in the “obstetric nephrology” field. Finally, situations of economic and cultural disadvantage, even in rich countries, contribute to increasing the rates of unplanned pregnancies.

Although the literature lacks comparative data on the outcomes for women who did or did not undergo pre-conception counseling, the most difficult situations arise precisely in the context of unplanned pregnancies, especially when these occur during the active phases of the disease or while taking drugs that should be avoided.

In cases of unplanned pregnancy, the management decision should be personalized and shared with the patient, considering various aspects not only related to renal disease but also encompassing gestational age, ovarian reserve, and the acceptability of a risk of malformation in cases otherwise deemed unacceptable [110].

### 8.2. Antihypertensive Therapy in Pregnancy

Although specific guidelines are lacking, recent evidence suggests optimal maximum values of 130/80 mmHg, with the eventual use of medications allowed in pregnancy (Table 4) [26,31]. Mainly, for pregnant women with hypertension without contraindications, first-line medications include labetalol, nifedipine, and alpha-methyldopa [26,31]. In emergencies, intravenous administration of labetalol, urapidil, and hydralazine is permitted [26]. ACE inhibitors (ACEi) and angiotensin receptor blockers (ARB) are contraindicated and should be discontinued [26,31]. The appropriate time to stop ARBs and ACE inhibitors is a debated issue, with some experts favoring adjustments post-confirmation of pregnancy to avoid prolonged discontinuation, while others recommend discontinuation before conception [26]. In this context, a personalized approach, considering renal function, severity of proteinuria, glomerular disease type, blood pressure control, and patient preferences, is fundamental for comprehensive and effective care.

### 8.3. Other Indications

The utilization of low-dose aspirin prophylaxis has become a standard practice for managing pregnancies at risk for preeclampsia (PE), encompassing situations involving women with glomerulonephritis [26,31]. Initiation of treatment at an early stage, preferably before the 12th week of gestation, is indicated [26]. The discontinuation of this prophylactic regimen is advised between the 34th and 36th weeks of gestation or in the presence of conditions at imminent risk for delivery [26].

Vitamin D deficiency, which is prevalent in advanced CKD, is linked to an increased susceptibility to preeclampsia, warranting meticulous monitoring and supplementation [26].

Prudent clinical management includes the avoidance of excessive gestational weight gain and adherence to low-protein diets, especially in cases of advanced CKD accompanied by substantial proteinuria [26].

In instances of severe hypoalbuminemia, interventions such as compression stockings and potential thrombo-prophylaxis with anticoagulants may be considered.

Table 5 summarizes the management of the main aspects of pregnancy in CKD women according to European guideline best practices.

**Figure 1:**
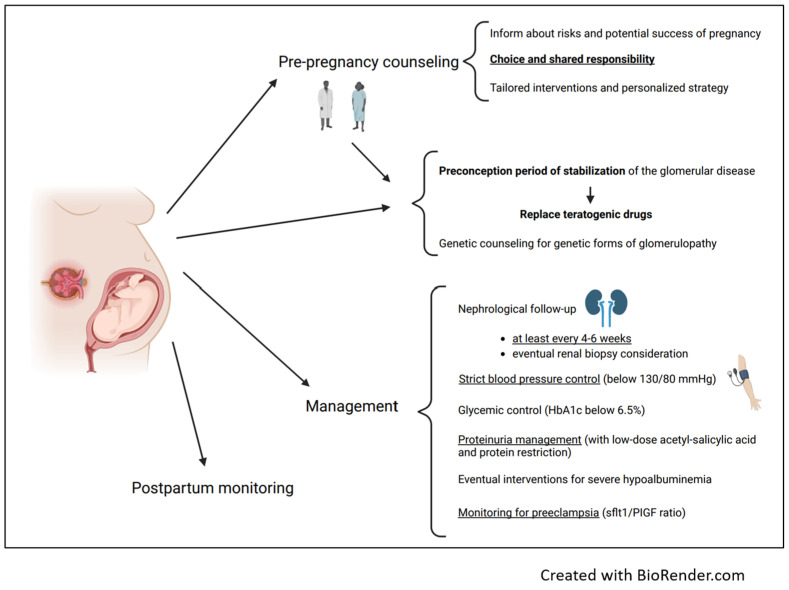
Counseling and management in women affected by glomerulonephritis who want to become pregnant.

In Figure 1, we summarize the main actions of counseling and management in women affected by glomerulonephritis who want to become pregnant. 

## 9. Limits of the Studies and Implications for Future Research

The paramount constraint in deriving definitive conclusions within the context of pregnancy in women afflicted with glomerulonephritis stems from the conspicuous absence of randomized controlled clinical trials, primarily attributable to ethical reservations. The existing recommendations hinge predominantly on retrospective observational studies or, to a lesser extent, prospective studies featuring modest-sized cohorts. These investigations frequently pivot around the assessment of the stage of kidney disease, a parameter that may be influenced by physiological hyperfiltration, and often employ definitions that lack uniform consensus. Specific etiological factors, such as proteinuria, hypertension, and the presence of genetic or infectious disorders, are not consistently factored into these analyses, thereby complicating a lucid evaluation of outcomes across diverse glomerulopathies. Furthermore, the challenge persists in aggregating numerically sufficient patient cohorts across disparate studies, a requisite for achieving statistical significance post-stratification of individual risk factors. Future strides in research should rigorously address these limitations by championing meticulously designed randomized controlled trials augmented by more expansive cohorts. These endeavors must incorporate standardized definitions and consider specific causative factors to increase clarity and robustness in delineating conclusions within the intricate landscape of pregnancy in glomerulonephritis.

## 10. Conclusions

Women with glomerulopathy confront an elevated risk of encountering complications throughout the course of pregnancy. This risk is particularly heightened when the glomerular disease is accompanied by classic outcome modifiers such as renal dysfunction, proteinuria, and hypertension. It is worth emphasizing that the available studies on this matter do not lend themselves to precise conclusions; however, rather than absolute contraindication, data from recent years lean toward a proactive stance. Planning and managing the pregnancy become critical components of care. This includes thoroughly identifying potential risks, informing the woman about these risks, and implementing the most effective means to ensure the best possible outcome for both the mother and the child. Multidisciplinary care, involving collaboration among various medical specialists, is also paramount. Essentially, until well-conducted studies yield conclusive results, it is imperative to adopt a careful approach to minimize risks and enhance outcomes. 

## Figures and Tables

**Table 2:** Safety in pregnancy on the immunosuppressive drugs.

Drugs	Safety According to Food Drug Administration
Allowed	
Steroids	C (risk cannot be ruled out)
Azathioprine	D (positive evidence of risk)
Hydroxychloroquine	B (no evidence of risk in humans)
Tacrolimus	C (risk cannot be ruled out)
Cyclosporine	C (risk cannot be ruled out)
Insufficient evidence	
Cyclophosphamide	D (positive evidence of risk)
Rituximab	C (risk cannot be ruled out)
Eculizumab	C (risk cannot be ruled out)
Intravenous Immunoglobulins	C (risk cannot be ruled out)
To be avoided	
Methotrexate	X (contraindicated in pregnancy)
Mycophenolate Mofetil	D (positive evidence of risk)
Sirolimus and Everolimus	C (risk cannot be ruled out)

**Table 3:** Immunosuppressive treatment options in glomerulonephritis during pregnancy [26].

MCD/FSGS	MN	IgAN	LN
CS	CNI	CS	HCQ
CNI		CNI	CS
AZA		AZA	AZA
			CNI

Legend: MCD, minimal change disease; FSGS, focal segmental glomerular sclerosis; MN, membranous nephropathy; IgAN, IgA nephropathy; LN, lupus nephritis; CS, corticosteroids; CNI, calcineurin inhibitor; HCQ, hydroxychloroquine; AZA, azathioprine.

**Table 4:** Safety in pregnancy on the anti-hypertensive drugs.

Drugs	Safety According to Food Drug Administration
Allowed—First choice	
Alpha-methyl dopa	B (no evidence of risk in humans)
Niphedipine	C (risk cannot be ruled out)
Labetalole	C (risk cannot be ruled out)
Allowed—Second choice	
Atenolole	D (positive evidence of risk)
Pindolole	B (no evidence of risk in humans)
Metoprolol	C (risk cannot be ruled out)
Clonidine Alpha blockers	C (risk cannot be ruled out) C (risk cannot be ruled out)
To be avoided	
Short acting niphedipine	D (positive evidence of risk)
ACE-I and ARB	C (risk cannot be ruled out) 1st trimester D (positive evidence of risk) 2nd 3rd trimester

**Table 5:** Management of blood pressure, preeclampsia, and immunosuppressive drugs for pregnancy in CKD according to European guidelines-best practices.

Guidelines	Blood Pressure		Preeclampsia				Immunosuppressive Drugs
			Role of Biomarkers	Indications for Acetylsalicylic Acid Administration		Other Medications	
	Target Pressure	Antihypertensive Therapy		To Who?	Dose		
Italian best practices (2016) [31]	Ideal: <130/80 mmHg Acceptable: <140/90 mmHg	FIRST CHOICE drugs: α-methyldopa Niphedipine Labetalole SECOND CHOICE drugs: β-blockers Clonidine α-blockers To be AVOIDED: Short acting niphedipine ACEi, ARB and related drugs	For the differential diagnosis between CKD and PE, the sFlt-1/PIGF ratio is considered one of the most promising predictors of PE.	Subject of ongoing debate. Current evidence supports acetylsalicylic acid use in high risk of PE populations. For patients with SLE, high antiphospholipid antibody titers, or triple antibody positivity, low-molecular-weight heparin with or without low-dose aspirin should be used.	Low-dose	No evidence supports the use of vitamin D supplementation to reduce adverse pregnancy outcomes, including PE.	RELATIVE SAFE: Azathioprine Cyclosporine A Tacrolimus Steroids Hydroxychloroquine To be AVOIDED: Cyclophosphamide Mycophenolate Rituximab
British guidelines (2019) [106]	≤135/85 mmHg	SAFE: Labetalol Nifedipine Amlodipine Methyldopa Doxazosin Hydralazine β-blockers UNSAFE: ACEi (Continue until conception if required for nephroprotection) ARB Thiazide diuretics	A role for angiogenic markers (PlGF ± sFlt-1) is suggested in the diagnosis of superimposed PE, although further evidence on their efficacy is needed.	Acetylsalicylic acid decreases risk of PE in the general obstetric population included women with CKD.	75–150 mg (insufficient data on optimum dose 75 vs. 150)	Calcium supplementation’s impact on PE remains uncertain. Considering potential cardiovascular risks associated with positive calcium balance in CKD women, the guideline advises against calcium supplementation to mitigate pre-eclampsia risk, based on the current evidence. Oral vitamin D supplementation seems to reduce PE, low birth weight, and preterm birth risks. Optimal serum calcifediol (25(OH)-vitamin D) levels and cholecalciferol/ergocalciferol doses are unknown. Guidelines recommend monitoring calcifediol levels, offering replacement therapy (cholecalciferol 20,000 IU per week) until > 20 ng/mL (>50 nmol/L). Upon restoring calcifediol levels, continue activated vitamin D analogues (alfacalcidol, calcitriol) during pregnancy at maintenance doses. Women with CKD not needing activated analogues may receive a daily vitamin D maintenance dose (400–1000 IU) during pregnancy, based on ethnicity and BMI.	SAFE: Corticosteroids Hydroxychloroquine Azathioprine Ciclosporin Tacrolimus UNSAFE: Mycophenolate mofetil (contraception during treatment and for 6 weeks after treatment) Cyclophosphamide (contraception during treatment and for 3 months after treatment) Sirolumus/Everolimus (contraception during treatment and for 3 months after treatment) UNCLEAR: Rituximab (treatment decision depends on indication and alternative options) Eculizumab (treatment decision depends on indication and alternative options)
Dutch guidelines (2022) [107]	Preconceptionally <130/80 mmHg; During pregnancy, for patients in antihypertensives treatment, range is between 130/80 mmHg and 140/90 mmHg; After birth ≤130/80 mmHg.	SAFE: Methyldopa β-blocking agents α and β-blocking agents (labetalol) Lipophilic β-blocking agents (metoprolol, bisoprolol) Lipophilic β-blocking agents with partial agonist activity (pindolol) Dihydropyridine calcium antagonists in 2nd and 3rd trimester (limited evidence for safety in 1st trimester) Loop and thiazide diuretics can be continued during pregnancy if used preconceptionally; start only with strict indication in 2nd half of pregnancy UNSAFE: Triamterene Spironolactone Renin-angiotensin inhibitors (ACEi, ARB, renin inhibitors) (until 8 weeks amenorrhoea probably safe, but after teratogenic and fetotoxic). UNCLEAR: Hydrophilic β-blocking agents (atenolol, sotalol) Amiloride (very limited data for safety in 1st trimester)	Do not rely on the sFlt-1/PIGF ratio in routine clinical practice for distinguishing between PE and underlying CKD, as the currently available data are not yet deemed sufficiently reliable.	Prescribe acetylsalicylic acid to any pregnant woman with CKD because she has a higher risk of PE.	80–150 mg/day. Starting from 12 weeks and preferably before the end of the 16 weeks. Stop treatment at least 1 week before expected natural birth or scheduled caesarean section.	Advise pregnant women with CKD to consume at least 1000 mg/day of elemental calcium, preferably from food, to potentially lower the risk of PE. If dietary calcium intake is insufficient initiate elemental calcium supplementation with 500–1000 mg (preferably, use a combination of calcium and 400–800 IU of cholecalciferol). Do not recommend sodium or protein restriction specifically for preventing PE in pregnant women with CKD.	SAFE: Corticosteroids Hydroxychloroquine Azathioprine Tacrolimus IVIG Ciclosporine Eculizumab CONTROINDICATED: Mycophenolate mofetil (discontinue at least 3 months before conception) Methotrexate (discontinue at least 3 months before conception) DISCOURAGED: Cyclofosfamide (use only with strict indication in 2nd or 3rd trimester) Rituximab (discontinue at least 6 months before conception) (use only when non safer alternative available) UNKNOWN RISK: Alemtuzumab Belatacept Belimumab rATG
German guidelines (2022) [108]	Range is between 110/70 mmHg and 135/85 mmHg	SAFE: α-methyldopa (1st choice) Nifedipine retard Amlodipine UNSAFE: Diuretics ACEi ARB UNCLEAR: Urapidil Selective β1-blockers (metoprolol)	If there is a suspicion of PE or PE cannot be excluded, the sFlt-1/PIGF ratio can be used to exclude or confirm a diagnosis of PE.	Pregnant women with CKD.	150 mg of acetylsalicylic acid for PE prophylaxis during weeks 12–36. For pregnant women with antiphospholipid syndrome lacking previous thrombosis history prescribe a combined treatment of 100 mg acetylsalicylic acid to and prophylactic doses of heparin. If there’s a prior history of arterial or venous thrombosis, administer therapeutic doses of heparin.		SAFE: Azathioprine Cyclosporine A Hydroxychloroquine Tacrolimus UNSAFE: Mycophenolate mofetil Cyclophosphamide Leflunomide UNCLEAR: Eculizumab Rituximab

Legend: ACEi, Angiotensin converting enzyme inhibitors; ARB, Angiotensin receptor blockers; CKD, chronic kidney disease; PE, preeclampsia; sFlt-1, soluble fms-like tyrosine kinase-1; PIGF, placental growth factor; SLE, systemic lupus erythematosus; IVIG, intravenous immunoglobulins; rATG, rabbit anithymocyte globulin.

## Data Availability

Not applicable.
